# Metabolomic Perspectives in Antiblastic Cardiotoxicity and Cardioprotection

**DOI:** 10.3390/ijms20194928

**Published:** 2019-10-04

**Authors:** Martino Deidda, Valentina Mercurio, Alessandra Cuomo, Antonio Noto, Giuseppe Mercuro, Christian Cadeddu Dessalvi

**Affiliations:** 1 Department of Medical Sciences and Public Health, University of Cagliari, 09042 Monserrato-Cagliari, Italy; martino.deidda@tiscali.it (M.D.); giuseppemercuro@gmail.com (G.M.);; 2 Department of Translational Medical Sciences, Federico II University, 80131 Naples, Italy; valentina.mercurio@unina.it (V.M.); alebcuomo@gmail.com (A.C.)

**Keywords:** metabolomics, cardiotoxicity, heart failure, metabolism

## Abstract

Despite advances in supportive and protective therapy for myocardial function, cardiovascular diseases due to antineoplastic therapy—primarily cardiomyopathy associated with contractile dysfunction—remain a major cause of morbidity and mortality. Because of the limitations associated with current therapies, investigators are searching for alternative strategies that can timely recognise cardiovascular damage—thus permitting a quick therapeutic approach—or prevent the development of the disease. Damage to the heart can result from both traditional chemotherapeutic agents, such as anthracyclines, and new targeted therapies, such as tyrosine kinase inhibitors. In recent years, metabolomics has proved to be a practical tool to highlight fundamental changes in the metabolic state in several pathological conditions. In this article, we present the state-of-the-art technology with regard to the metabolic mechanisms underlying cardiotoxicity and cardioprotection.

## 1. Introduction

Drug-induced cardiotoxicity (CTX) can seriously affect patient survival during anticancer therapy. In recent years, CTX has become one of the main topics of investigation aimed at reducing the burden of morbidity in long-term cancer survivors.

Anthracyclines remain the most commonly studied CTX drugs [1]; just recently, new interpretations of their cardiotoxic effect in association with new targeted therapies have been advanced [2]. Furthermore, many other anticancer drugs have more recently shown a significant CTX, mostly caused by their joint action on both the signalling pathways required for tumour growth and those involved in normal cardiovascular function [3].

Since conventional CTX indexes often show appreciable changes only after cardiac damage has occurred, increasing efforts have been made to find new diagnostic tools [4]. Accordingly, in recent decades, metabolomic research has focused on the mechanisms of CTX, identifying new signalling pathways and proposing new possible biomarkers of cardiac damage, precocious enough to precede clear clinical manifestations. [5] This approach, successfully used in studying metabolic disorders occurring in heart failure (HF) [6,7]—which is also the most studied complication of anti-cancer therapy—could contribute significantly to increasing our knowledge in the CTX setting. 

## 2. Metabolic Derangements in Heart Failure (HF)

Anthracycline-induced HF constitutes the historically most common form of antineoplastic drug-induced CTX. Metabolomics findings suggest that energy metabolism is a critical target in the development of this CTX form. Therefore, researches aimed to investigate these pathways are needed for the identification of early markers of CTX and the development of innovative cardioprotective agents [5]. Nevertheless, we acknowledge that the actual protective role of treatments targeting these pathways in the prevention of CTX still needs to be demonstrated.

In this context, metabolomics could significantly help improve the knowledge of these alterations and identify new therapeutic targets that could modify the natural progression of HF and improve survival in patients with this disease.

Cardiomyocytes′ energetic metabolism is a crucial feature of myocardial contractile function. Three components define it: substrate uptake and utilisation (using beta-oxidation and glycolysis), energy production (mitochondrial oxidative phosphorylation), and energy transfer and utilisation (ATP consumption by the creatine kinase energy shuttle). Alterations in those components may have a pivotal role in the pathophysiology of HF [8]. Furthermore, the modulation of these metabolic alterations has been proposed as a promising new target for its therapy.

Here, we review the main alterations in cardiac energy metabolism and describe the implications of these alterations for the treatment of HF, understood as the most common setting of antiblastic therapy CTX.

Cardiomyocytes’ substrate utilisation involves the consumption of free fatty acids (FA) and glucose. Both substrates are metabolised to acetyl coenzyme A, through beta-oxidation or glycolysis, respectively. In a failing heart, a reduction in both the uptake and the metabolisation of these substrates has been described. In particular, some studies have demonstrated that FA consumption is reduced in advanced HF [9] but seems to be unchanged or slightly increased in the early stages of the disease [10]. Interestingly, glucose consumption is increased in the first stages of HF [11], while it progressively decreases in advanced HF and is associated with the development of insulin resistance [12].

PDH kinase inactivates pyruvate dehydrogenase (PDH), but high levels of pyruvate can inhibit PDH kinase activity. In a healthy heart, PDH has a pivotal role in the production of acetyl-CoA, destined to the Kreb’s cycle, while its function is impaired in HF. Moreover, there is a tight connection between glycolysis and FA oxidation and PDH activity in glucose oxidation. In HF, mitochondrial activity is impaired, with compensatory activity of glycolysis and higher production of lactic acid that is additionally harmful for the already failing heart. Accordingly, the administration of dichloroacetate (DCA), a pyruvate analogue, increases glucose oxidation [13] but can also lead the pyruvate metabolism into acetyl-CoA instead of lactic acid.

Lately, many studies have focused on the role of carnitine palmitoyl transferase I (CPTI) and its possible inhibition to delay the onset of HF. CPTI transports FA in the cytosol to the mitochondria; drugs that inhibit CPTI, such as perhexiline, not only can reduce long-chain FA uptake but also can increase the activation of pyruvate dehydrogenase and, therefore, glucose oxidation. [14,15] Some drugs commonly used for the treatment of arrhythmias, such as amiodarone, can partially inhibit CPTI; this effect may partly contribute to explaining their beneficial role in HF [16]. Another target for novel therapeutic approaches to HF could be a partial inhibition of FA oxidation. It is noteworthy that some drugs already approved for cardiovascular disease (CVD) seem to be able to guarantee this effect. In particular, trimetazidine, an anti-anginal drug, can alter β-oxidation by inhibiting long-chain 3-ketoacyl-CoA thiosome (LC 3-KAT) [16,17]. Trimetazidine may also slightly inhibit CPTI [18] and improve both diastolic and systolic functions, as well as reduce reactive oxygen species (ROS) levels, improving the overall metabolic status of cardiomyocytes [19]. Similar effects have been reported for ranolazine [16]. Another important enzyme in cardiomyocyte metabolism is malonyl-CoA decarboxylase (MCD), which transforms malonyl-CoA, a natural CPTI inhibitor, into acetyl-CoA. Inhibition of MCD can potentially be a new target for HF [13], as it can also stimulate glucose oxidation, increasing PDH activity [20,21].

The second pathway that can be altered in HF is the oxidative phosphorylation of ADP by ATP-synthase. In fact, in HF, there is an impairment of mitochondrial function, including the respiratory chain, with the consequent reduction of ATP production through ADP phosphorylation [22]. A fundamental regulator of oxidative metabolism is proliferator-activated receptor-γ (PPAR-γ) coactivator 1α (PGC-1α). PGC-1α has been shown to increase mitochondrial respiratory chain activity by direct stimulation as well as by stimulation of FA oxidation [23]. In particular, mitochondria from failing cardiomyocytes have defects in their DNA due to increased production of ROS [24]; they also show a reduction in PPAR-γ and PGC-1α levels, with further worsening of mitochondrial activity [25,26]. Administration of adenoviral ectopic PGC-1α has been shown to improve oxidative respiration in enzyme-knock-out mice. Further studies will be needed to demonstrate whether the increase in PPAR-γ or PGC-1α activity could be a valid strategy to treat HF [27].

In an animal model of HF, the administration of fenofibrate, a PPARα agonist, led to a reduction in the levels of brain natriuretic peptide (BNP) and ROS production [28]; the treatment also showed an increase in the oxidation of FA [29].

Moreover, in cellular and animal models of myocardial ischemia and HF, a relevant alteration in the expression of some mitochondrial uncoupling proteins (UCP), such as UCP2 and UCP3, has been described [30]. This finding was associated with greater heat production, to the detriment of ATP synthesis, especially in the presence of increased FA levels and reduced glucose transporter 4 levels. [31] Therefore, the reduction of free FA levels in patients with HF could be a good therapeutic target to decrease the activity of UCPs and partially restore the synthesis of ATP in the heart [8].

The energy transfer through the creatine kinase (CK) shuttle is another pathway compromised during HF. Physiologically, the transfer of the phosphoryl group between phosphocreatine (PCr) and ATP mediated by CK is about 10 times faster than the production of new ATP by the oxidative phosphorylation. PCr is smaller than ATP in terms of molecular mass, thus its cellular concentration is higher than that of ATP, and it represents the energy reserve of muscle cells: whenever more energy is needed, the concentration of PCr decreases to keep the level of ATP constant. In a healthy heart, PCr assures high level of energy are easily available and, since its molecular size is smaller than that of ATP, easily storable in the cell [32]. In HF, there is an increase in required energy, as in the consumption of ATP. The resulting increase in ADP levels can inhibit many intracellular enzymes. Besides, there may be a further decrease in the ADP cell reserve, due to the use of two molecules of ADP to produce one molecule of AMP and one molecule of ATP. AMP can easily cross the cell membrane, leading to a decrease in the adenosine cell reserve; this contributes to the impairment of the cellular contractile system, with a reduction of the inotropic reserve of the heart [8,33]. Furthermore, it appears that the energy transfer from CK to ATP is impaired with an alteration of the PCr/ATP ratio [34]. In particular, while the concentration of ATP is initially stable in HF and decreases by 30–40% only in advanced HF, [35,36], the concentrations of creatine and PCr are reduced by 30–70% since early stages of the disease [37,38].

The stimulation of AMP kinase by indirect activators such as 5′-aminoimidazole-4-carboxyamide-ribonucleoside (AICAR) or metformin, can be used as a potential new therapeutic strategy [39].

The activation of adenosine-monophosphate kinase (AMPK) interferes with the metabolism of both glucose and FA. It leads to an increase of intracellular glucose levels by activating glucose transporters (GLUTs) and stimulating gluconeogenesis and glycolysis. The activated AMPK can also stimulate the synthesis, absorption, and oxidation of FA, thus reducing the concentrations of malonyl-CoA [40]. Metformin has a well-known positive effect on cardiac function, reducing cardiomyocytes apoptosis and ROS production, slowing HF progression [41] and improving the synthesis of ATP by the mitochondrial respiratory chain [42].

**Figure 1:**
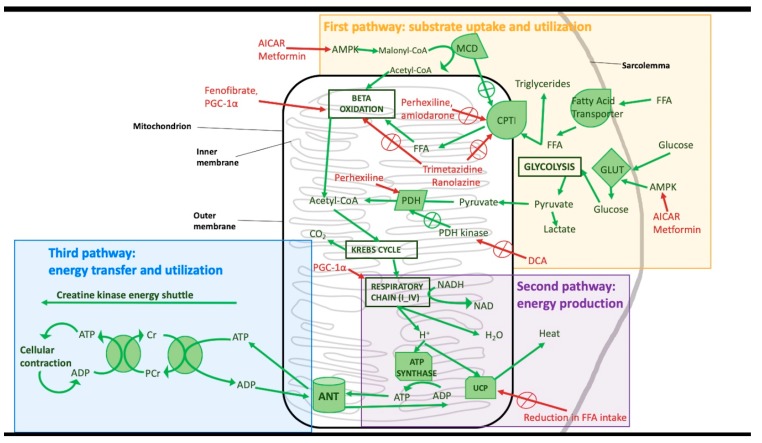
Metabolic derangements in the failing myocardium. AICAR: 5′-aminoimidazole-4-carboxyamide-ribonucleoside; MCD: malonyl-CoA decarboxylase; PGC-1α: proliferator-activated receptor-γ coactivator 1α; CPTI: carnitine palmitoyl transferase I; FFA: free fatty acids; GLUT: glucose transporters; PDH: pyruvate dehydrogenase; PCr: phosphocreatine, ANT: Anthracyclines, such as Doxorubicin; UCP: mitochondrial uncoupling proteins.

Figure 1 summarises the alterations in cardiac energy metabolism described above and the possible therapeutic targets.

## 3. Experimental Techniques Used in Metabolomics Studies

Metabolomics is the measurement of multiple small-molecule metabolites in biological samples that include all body fluids, tissues and exhaled breath [43]. The metabolomic analysis uses analytical technologies, which are able to identify low-molecular-weight molecules such as nucleic acids, vitamins, amino and FA, organic acids, carbohydrates, lipids, polyphenols and inorganic and elemental species. Human biological samples provide some metabolites that depend on the specimen under examination and the used analytical technique. The Human Metabolome Database [44] stores over 40,000 metabolites, identified, quantified and catalogued by the Human Metabolome Project [45].

The metabolomic approach consists of three sequential steps: (1) samples collection and storage, (2) samples analysis using an experimental technique, (3) data analysis.

About the first step, the following rules should always be applied: collection of samples in a sterile container containing micromolar quantities of inorganic bacteriostatic agents such as sodium azide (0.01–0.1%) to avoid metabolic alterations due to bacterial metabolism; quick centrifugation of the samples at high speed to eliminate cellular debris, including active enzymes that can modify the metabolic content; early freezing of the samples at very low temperatures (−40 °C, −80 °C), up to the analysis; thawing the samples on ice to avoid rapid and harmful temperature variations [46].

Regarding the second step, among the many different technological platforms, nuclear magnetic resonance (NMR) spectroscopy and mass spectrometry (MS) are the most used (Table 1) [47]. The NMR technique can simultaneously detect, in a non-targeted manner, signals originating from many different molecules (amino acids, organic and FA, lipids, carbohydrates and amines) without sample pre-treatment, thus providing a molecular “snapshot” of the biological sample. On the other hand, MS, combined with gas chromatography (GC) or liquid chromatography (LC) which act as separation techniques, identifies the molecules of the analysed samples on the basis of their mass-to-charge ratio. Each resulting peak is originated from the characteristic mass spectrum of the metabolite, allowing its identification. MS deals with the measurement of selected extracted metabolites. Its advantage is higher sensitivity compared to NMR (nM vs. mM); however, it always requires manipulation of the samples and time-consuming extraction and derivatisation procedures that irreversibly modify the samples under investigation. 

Finally, data analysis and interpretation produce complex data matrices, consisting of the quantitative measurement of several hundred metabolites. All the obtained data are subsequently analysed with chemometrics methods, multivariate statistical tools that allow the extraction of biological, physiological and clinical information. Before statistical analysis, the data are subjected to several processing steps: (1) processing of the different spectra, according to the analytical techniques used; (2) production of a data matrix with m rows (observations, samples) and n columns (variables, frequencies, integrals); (3) data normalisation and scaling [48]; (4) multivariate statistical data modelling.

The complexity of the spectroscopic data derived from the commonly high number of samples and variables (i.e., metabolites or instrumental parameters) necessarily requires the application of data reduction techniques to extract latent metabolic information and allow sample classification and the identification of a potential biomarker. With this aim, both uni- and multivariate analyses can be applied.

## 4. Metabolomic Studies in Cardiotoxicity and Cardioprotection

Table 2 reports the results of the discussed studies.

Andreadou et al. performed a pioneering NMR metabolomics profile of acute doxorubicin (DOX)-induced CTX in rats. Three days after DOX administration, a time sufficient to determine acute CTX at the cardiomyocyte level, as previously demonstrated [49], the authors removed the hearts and acquired ^1^H-NMR spectra of aqueous myocardial extracts. Myocardial levels of acetate and succinate increased in DOX-treated samples, while branched-chain amino acids decreased. The authors concluded that acetate and succinate could be useful as CTX biomarkers and that oleuropein, a phenolic antioxidant present in the olive tree with documented cardioprotective effects, could reduce the energy metabolic pathways distress [50].

The same group of researchers confirmed the role of oleuropein in the prevention of CTX in a study in which the metabolomic data were analysed together with cardiac geometry and function evaluated by echocardiography, cardiac histopathology, nitroxidative stress, inflammatory cytokines, NO homeostasis (iNOS and eNOS expressions), and kinases involved in apoptosis (Akt, AMPK). In the DOX group, the authors found reduced fractional shortening and left ventricular wall thickness on echocardiographic examination, an imbalance between the iNOS and eNOS expressions and disturbed protein biosynthesis. Furthermore, perturbations of energy metabolism were identified. Oleuropein-induced AMPK activation and iNOS suppression seemed to be able to prevent the negative cardiac effects of DOX. [50] Noteworthy, in both the studies of Andreadu, the identified metabolites resulted related to energy production pathways [50,51].

A GC–MS metabolomic study confirmed the centrality of perturbed energetic metabolism in the development of CTX, evaluated as increases in CK, creatine kinase-MB (CK-MB) and lactate dehydrogenase (LDH) 72 h after DOX injection. Tan and al. identified a fingerprint in this rat model of DOX-induced CTX, consisting of 24 metabolites involved in glycolysis, citrate cycle and metabolism of some amino acids and lipids [52].

A liposomal drug delivery system was developed to reduce the cumulative CTX of the anthracycline pirarubicin (THP). On this basis, Cong and colleagues studied the urine metabolic footprint of Sprague-Dawley rats after three successive doses of liposome powder THP (L-THP) or free THP (F-THP); CTX was clinically evaluated by statistically significant bodyweight reduction in treated rats compared to control animals and confirmed on the basis of the results of heart samples histopathology tests. The metabonomic analysis showed that L-THP determined only minimal metabolic changes compared to F-THP; furthermore, the study showed that subsequent doses of THP led to severe metabolic alterations, particularly at the level of energy production pathways. In detail, citrate (Krebs cycle), lactate (glycolysis), d-gluconate-1-phosphate (pentose phosphate), *N*-acetyl glutamine and *N*-acetyl-dl-tryptophan (amino acid metabolism) were significantly downregulated in the treatment groups. It is noteworthy that a greater downregulation of the intermediate metabolites in the F-THP group compared to the L-THP group was observed [53].

A metabolomic analysis with ultra-performance liquid chromatography–quadrupole time-of-flight mass spectrometry (UPLC–Q-TOF-MS) in a CTX mouse model revealed 39 biomarkers capable of identifying CTX, defined as severe heart damage on the basis of the results of biochemical analysis and histopathological assessment. With the aim of filtering out the exclusive CTX biomarkers, the identified metabolites were processed for verification and optimisation combined with hepatotoxicity and nephrotoxicity, thus obtaining a panel consisting of 10 highly specific metabolites. Among them, the most strongly specific were l-Carnitine, 19-hydroxydioxycortic acid, lysophosphatidylcholine (LPC) (14:0) and LPC (20:2); it is noteworthy that the panel of identified metabolites were shown to change before biochemical and histopathological alterations were detected [54].

A mass spectrometry-based and NMR spectrometry-based metabolomic study designed to identify the early biomarkers of DOX-induced CTX (evaluated by differences in body weight gain during the treatment and confirmed by histopathology) was performed on male B6C3F1 mice. A 3 mg/kg DOX dose or saline were administered weekly for 2, 3, 4, 6 or 8 weeks to mice, sacrificed one week after the last dose. An increase of 18 amino acids and 4 biogenic amines (acetylornithine, kynurenine, putrescine and serotonin) was found at the myocardial level after a cumulative dose of 6 mg/kg. On the contrary, the myocardial lesion was detected only at 18 mg/kg of cumulative dose, and the cardiac pathology was identified at 24 mg/kg of cumulative dose. The metabolic analysis also found altered plasma levels of 16 amino acids and 2 biogenic amines (acetylornithine and hydroxyproline) and increased plasma levels of 16 acylcarnitines, whenever 5 acylcarnitines were decreased in cardiac tissue. It is noteworthy, that the plasma concentrations of the two intermediates of the Krebs cycle, lactate and succinate, were significantly altered after the cumulative dose of 6 mg/kg [55].

Rat plasma samples collected after cyclophosphamide (CY) administration were analysed using UPLC–Q-TOF-MS to identify possible biomarkers of CTX, defined by increasing in CK, CK-MB and LDH and confirmed by histopathological assessment. The authors found altered levels of a dozen plasma metabolites when comparing the CY-treated group after 1, 3 and 5 days with the control group. These results suggest that these substances, involved in the metabolism of linoleic acid and glycerol phospholipid, may be implicated in CY-induced CTX. On this basis, the authors suggested that CTX could result in increased myocardial oxygen consumption and impaired fatty acid β oxidation [56].

^1^H-NMR spectroscopy was employed to profile the culture medium of human induced pluripotent stem cell-derived cardiomyocytes (hiPSC-CMs) exposed to DOX for 2 days or 6 days in a repeated way. While a single exposure to DOX did not cause alterations of the extracellular metabolites, repeated exposures resulted in a reduction in the utilisation of pyruvate and acetate, with an accumulation of formate. Furthermore, during the drug washout, a reversible effect and a restored utilisation by hiPSC-CMs were demonstrated for pyruvate, while formate and acetate showed an irreversible effect. The authors concluded that pyruvate, acetate and formate could be used as biomarkers of DOX-induced CTX; moreover, they hypothesised that the alterations of these metabolites could be linked to ATP depletion and mitochondrial and that the latter may be the main cause of the observed CTX [57].

A pairwise comparative metabolomic study was conducted to identify a possible signature of both DOX-induced CTX and cardioprotection by dexrazoxane (DZR); the clinical sign used to determine the moment to euthanise the animals was weight loss >20%. For this purpose, 96 BALB/c mice, randomly divided into two groups (tumour and control), each subdivided into four treatment subgroups (control, DOX, DZR and DOX plus DZR) were used. DOX administration was characterised by an increase in 5-hydroxylysine, 2-hydroxybutyrate, 2-oxoglutarate and 3-hydroxybutyrate levels and a decrease in glucose, glutamate, cysteine, acetone, methionine, aspartate, isoleucine and glycylproline levels. On the other hand, treatment with DZR produced an increase in lactate, 3-hydroxybutyrate, glutamate and alanine levels and a decrease in glucose, trimethylamine *N*-oxide and carnosine levels. It is important to highlight that the authors concluded that their results seem to validate the altered energy metabolic profile present in CTX [58].

In recent years, tyrosine kinase inhibitors (TKI) have proven to be an effective therapy for a wide range of malignancies. This class of drugs also showed CTX, probably related to their deleterious impact on specific cardiac metabolic pathways, with which TKIs interact. A non-targeted GC–MS metabolomics approach was used to analyse the heart, skeletal muscle, liver and plasma collected from FVB/N mice (10 per group) treated with sorafenib or vehicle control every day, for two weeks. In sorafenib-treated mice, compared to controls, echocardiography showed CTX-induced systolic dysfunction, while metabolomic analysis revealed significant alterations in 11 metabolites, including a markedly altered taurine/hypotaurine metabolism [59].

Another study designed to investigate TKI-induced CTX was conducted on female FVB/N mice (10/group) treated with sunitinib (40 mg/kg), erlotinib (50 mg/kg) or vehicle (control group) daily for two weeks. The authors performed a non-targeted GC–MS metabolomic analysis on the heart, skeletal muscle, liver and serum. Compared to the control group, the sunitinib-treated mice showed a significant decrease in systolic function at echocardiography and significant decreases in docosahexaenoic acid, arachidonic acid/eicosapentaenoic acid, *O*-phosphocolamine and 6-hydroxynicotinic acid levels in a metabolomic analysis. On the other hand, erlotinib did not cause systolic dysfunction and only increased the metabolite spermidine. The authors concluded that their study highlighted the link between sunitinib-induced CTX, depletion of polyunsaturated FA and inflammatory mediators [60].

More recently, Yoon and colleagues carried out an ^1^H-NMR-based metabolomics study, evaluating the cardioprotective effects of spinochrome D (SpD) against 24/48 h exposure to 0.1 μM DOX in human cardiomyocytes and human breast cancer cells. SpD seemed to protect AC16 cells from Dox toxicity, without any effect on its anticancer properties. Moreover, SpD treatment determined different mitochondrial membrane potentials and calcium localisation between cardiomyocytes and cancer cell lines, suggesting a possible role of SpD against DOX-induced CTX. Twelve discriminating metabolites (decrease of acetate, glutamine, serine, uracil, glycerol; increase of glutamate, isoleucine, O-phosphocholine, taurine, myo-inositol, glutathione, sn-glycero-3-phosphocholine) were identified, and glutathione metabolism seemed to be the most significantly influenced pathway by SpD treatment [61].

Also, the exposure of the heart to ionising radiation can induce CTX, which, in turn, seems to be associated with metabolic changes in cardiac cells damaged by radiation. In 2018, Gramatyka and colleagues studied the metabolic response to radiation of human cardiomyocytes using high-resolution magic-angle-spinning nuclear magnetic resonance techniques (HR-MAS NMR). NMR spectra showed changes in lipids, threonine, glycine, glycerophosphocholine, choline, valine, isoleucine and glutamate, as well as reduced glutathione and taurine metabolism. These findings suggested that ionising radiation could influence the metabolic pathways of cardiomyocytes even at low doses, which potentially do not affect cell viability [62].

## 5. Conclusions

In conclusion, metabolomics demonstrated to be effective in identifying altered metabolic pathways in CTX; noteworthy, most of the studies seem to point out the alterations in energy metabolism as the most affected in the setting of antiblastic drug-induced CTX.

**Figure 2:**
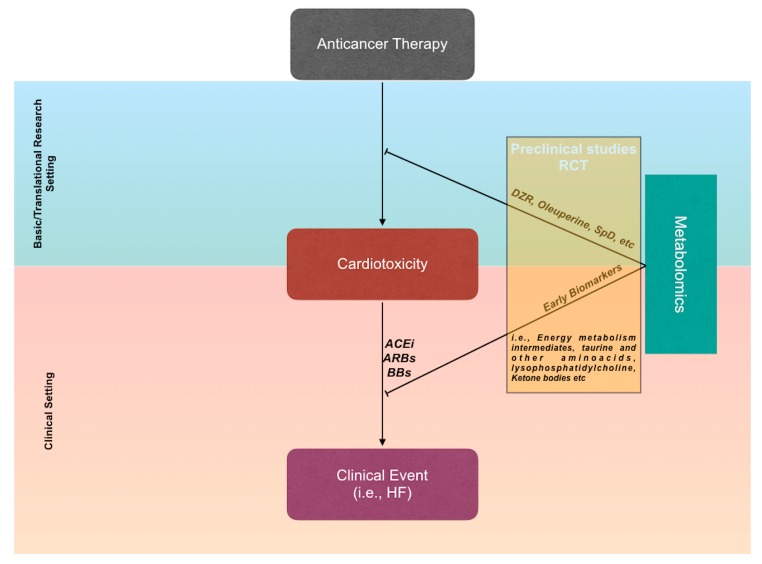
Future challenges of metabolomic studies. Translational research could accelerate the development of cardioprotective agents to be introduced rapidly in clinical practice. On the other hand, the identification of metabolite clusters associated with the risk of developing cardiotoxicity (CTX) could allow an early therapeutic intervention, before the occurrence of clinical events. Abbreviations: RCT: randomised clinical trials; ACEi: ACE-inhibitors; ARBs: angiotensin receptor blockers; BBs: beta-blockers; HF: heart failure.

The basic and translational metabolomic approach, identifying specific metabolic profiles associated with the risk of developing CTX, will make a priori stratification and very early identification of the CTX risk possible, well before the onset of significant changes reported by commonly used biomarkers, which are—almost all—indexes of occurred cardiac damage. Indeed, studies on animal models seem ready to translate this initial perception of efficacy into clinical models. Furthermore, it is desirable that the large number of pathophysiological data provided by metabolomics, as well as by other unconventional strategies [63,64], allow the identification of highly effective and individualised therapeutic strategies, able to prevent and treat CTX [65] (Figure 2).

## Figures and Tables

**Table 1:** Analytic techniques used in metabolomic studies (adapted from Deidda et al. [6]).

Technique	Cost	Throughput	Advantages	Disadvantages
High-resolution NMR spectroscopy (NMR)	Low per sample	~10 min	Simultaneous detection of many different compounds, such as carbohydrates, amino acids, organic and fatty Acids, amines and lipids without any initial sample pre-treatment Non-destructive technique Good libraries of spectra Easy to process	Poor sensitivity Co-resonances for 1D-NMR spectroscopy 2D-NMR spectroscopy is time-consuming
In vivo NMR spectroscopy	High	~30 min with preparation time	Possibility of observing the metabolism of the working heart Imaging can map metabolite distributions	Very poor sensitivity (hyperpolarization or higher field strengths could improve it)
High-resolution magic-angle-spinning NMR (HR-MAS-NMR)	Low	~15 min	Possibility of monitoring the cellular environment (e.g., compartmentation) in intact tissue Tissue can be chilled to reduce post-mortem effects	Tissue cannot be perfused, so its viability is limited
Direct-infusion MS	Low	3–4 min	Has been used to profile both aqueous and lipophilic metabolites in various studies Minimal carry-over, as no chromatography involved Good reproducibility Simple to optimise	Ion suppression can be a substantial problem; identification can require chromatography, e.g., for isobaric species; metabolite identification is a significant challenge and requires mass spectometry acquisitions; semiquantitative at best
GC–MS	Low-medium	20–30 min for FA 30–45 min for aqueous metabolites	Chromatography is robust and reproducible Metabolite identification is aided by the adoption of standard ionisation parameters in electron impact Can be quantitative	Metabolites need derivatisation, and not all metabolites are suitable for derivatisation
LC–MS	Medium	~15–30 min	Chromatography reduces the effect of ion suppression and can separate isobaric species Suitable for measuring intact lipids, dipeptides, tripeptides, and other macromolecules	Chromatography can drift during a sample run, which makes data processing difficult Metabolite identification is a major challenge
Triple quadrupole (targeted) MS	Medium to high	15 min per chromatography run ~60 min for more comprehensive screens	Highly sensitive Highly quantitative Targeted Results readily transferable because concentrations can be measured	Targeted, so the discovery of novel biomarkers is unlikely Time-consuming to set up quantitative assays

NMR: ^1^H nuclear magnetic resonance spectroscopy; HR-MAS-NMR: high-resolution magic-angle-spinning NMR; GC–MS: gas cromatography–mass spectrometry LC–MS: liquid cromatography–mass spectrometry; MS: mass spectrometry; WD: Western diet; LysoPC: lysophosphatidylcholine.

**Table 2:** Summary of techniques and results of the discussed studies.

Reference	Species	Technique	Biofluid/Tissue	Metabolites/Metabolism Discrimination
Andreadu et al., 2009 [50]	Wistar rats	NMR	Aqueous myocardial extracts	Increased levels of acetate and succinate, decreased levels of branched-chain amino acids
Andreadu et al., 2014 [51]	Wistar rats	NMR	Aqueous myocardial extracts	Perturbations of energy metabolism
Tan et al., 2011 [52]	ICR mice	GC–MS	Myocardial tissue	Increased levels of l-alanine, phosphate, glycine, succinate, malate, proline, threonic acid, glutamine, phenylalanine, dihydroxyacetonephosphate (DHAP), glycerol-3-phosphate (G-3-P), fructose, glucose, stearic acid, myo-inositol and cholesterol; decreased levels of lactate, β-hydroxybutyric acid, l-valine, isoleucine, threonine, citrate, linoleic acid, arachidonic acid
Cong et al., 2012 [53]	Sprague-Dawley rats	UPLC–TOF-MS	Urine	Metabolites involved in metabolic process related to myocardial energy metabolism: tricarboxylic acid cycle (citrate), glycolysis (lactate), pentose phosphate pathway (d-gluconate-1-phosphate) and amino acid metabolism (*N*-acetylglutamine and *N*-acetyl-dl-tryptophan)
Li et al., 2015 [54]	Wistar rats	UPLC–Q-TOF-MS	Plasma	l-carnitine, 19-hydroxydioxycortic acid, LPC (14:0) and LPC (20:2)
Schnackenberg et al., 2016 [55]	B6C3F1 mice	GC-MS, NMR	Heart tissue, Plasma	Myocardial specimens: altered levels of 18 amino acids and acetylornithine, kynurenine, putrescine and serotonin, decreased levels of 5 acylcarnitines. Plasma samples: altered levels of 16 amino acids and acetylornithine and hydroxyproline, increased levels of 16 acylcarnitines
Yin et al., 2016 [56]	Wistar rats	UPLC–Q-TOF-MS	Plasma	l-carnitine, proline, 19-hydroxydeoxycorticosterone, phuyoshingosine, cholic acid, LPC (14:0), LPC (18:3), LPC (16:1), LPE (18:2), LPC (22:5), LPC (22:6), linoleic acid, LPC (22:4), LPC (20:2), LPE (18:0), LPC (20:3)
Chaudhari et al., 2017 [57]	Human induced pluripotent stem cell-derived cardiomyocytes	NMR	Culture medium	Reduction in the utilisation of pyruvate and acetate, and accumulation of formate
QuanJun et al., 2017 [58]	BALB/c mice	NMR	Serum	DOX administration: increase in 5-hydroxylisine, 2-hydroxybutyrate, 2-oxoglutarate, 3-hydroxybutyrate decrease in glucose, glutamate, cysteine, acetone, methionine, asparate, isoleucine and glycylproline. DZR treatment: increase in lactate, 3-hydroxybutyrate, glutamate, alanine; decrease in glucose, trimethylamine *N*-oxide and carnosine levels
Jensen et al., 2017 [59]	FVB/N mice	GC–MS	Plasma and heart, skeletal muscle and liver tissues	Significant alterations in 11 metabolites, including markedly altered taurine/hypotaurine metabolism: glutamine, ethanolamine, stearamide, taurine, *O*-phosphocolamine, hypotaurine, myo-inosithol-2-phosphate, dehydroalanine, adenosine-5-monophosphate, glycerol-1-phosphate
Jensen et al., 2017 [60]	FVB/N mice	GC-MS	Serum and heart, skeletal muscle and liver tissues	Significantly lower heart and skeletal muscle levels of long chain omega-3 fatty acids docosahexaenoic acid (DHA), arachidonic acid (AA)/eicosapentaenoic acid (EPA) and increased serum O-phosphocholine phospholipid
Yoon et al., 2019 [61]	Human cardiomyocytes	NMR	Cardiomyocites	Decrease of acetate, glutamine, serine, uracil, glycerol; increase of glutamate, isoleucine, *O*-phosphocholine, taurine, myo-inositol, glutathione, sn-glycero-3-phosphocholine
Gramatyka et al., 2018 [62]	Human cardiomyocytes	HR-MAS NMR (High-Resolution Magic-Angle-Spinning Nuclear Magnetic Resonance)	Cardiomyocites	Lipids, threonine, glycine, glycerophosphocholine, choline, valine, isoleucine, glutamate; reduced glutathione and taurine metabolism

UPLC–TOF/MS: Ultra-performance liquid chromatography–time-of-flight mass spectrometry; UPLC–QTOF/MS: Ultra-performance liquid chromatography-quadrupole time-of-flight mass spectrometry; LPE: lysophosphatidylethanolamine.

## Abbreviations

CTX	Cardiotoxicity
ATP	Adenosine TriPhosphate
PDH	Pyruvate DeHydrogenase
DCA	DiChloreAcetate
CoA	CoEnzyme A
CPTI	Carnitine Palmitoyl Transferase I
FA	Fatty Acids
HF	Heart Failure
LC 3-KAT	Long-Chain 3-KetoAcyl-coA Thiolsase
ROS	Reactive Oxygen Species
MCD	Malonyl-CoA Decarboxylase
ADP	Adenosine DiPhosphate
PPAR-γ	Proliferator-Activated Receptor-γ
PGC-1α	Proliferator-activated receptor-γ Coactivator 1α
BNP	Brain Natriuretic Peptide
UCPs	Mitochondrial Uncoupling Proteins
AMP	Adenosine MonoPhosphate
CK	Creatine Kinase
AICAR	5′-AminoImidazole-4-CarboxyAmide-Ribonucleoside
AMPK	Adenosine-MonoPhosphate Kinase
GLUTs	Glucose Transporters
NMR	Nuclear Magnetic Resonance
MS	Mass Spectrometry
GC	Gas Chromatography
LC	Liquid Chromatograph
HR-MAS-NMR	High-resolution magic-angle-spinning NMR
DOX	Doxorubicine
iNOS	inducible Nitric Oxide Synthases
eNOS	endothelial Nitric Oxide Synthases
THP	pirarubicin
L-THP	Liposome pirarubicin
F-THP	Free pirarubicin
CY	Cyclophosphamide
UPLC–Q-TOF-MS	Ultra-Performance Liquid Chromatography–Quadrupole Time-of-Flight MS
^1^H-NMR	Hydrogen NMR
hiPSC-CMs	Human induced Pluripotent Stem Cell-Derived CardioMyocytes
DZR	Dexrazoxane
TKIs	Tyrosine Kinase Inhibitors
HR-MAS NMR	High-Resolution Magic-Angle-Spinning NMR

## References

[1] Mele D., Nardozza M., Spallarossa P., Frassoldati A., Tocchetti C.G., Cadeddu C., Madonna R., Malagù M., Ferrari R., Mercuro G. (2016). Current views on anthracycline cardiotoxicity. Heart Fail. Rev..

[2] Cadeddu C., Piras A., Dessì M., Madeddu C., Mantovani G., Scartozzi M., Hagendorff A., Colonna P., Mercuro G. (2017). Timing of the negative effects of trastuzumab on cardiac mechanics after anthracycline chemotherapy. Int. J. Cardiovasc. Imaging.

[3] Madeddu C., Deidda M., Piras A., Cadeddu C., Demurtas L., Puzzoni M., Piscopo G., Scartozzi M., Mercuro G. (2016). Pathophysiology of cardiotoxicity induced by nonanthracycline chemotherapy. J. Cardiovasc. Med. (Hagerstown).

[4] Mercuro G., Cadeddu C., Piras A., Dessì M., Madeddu C., Deidda M., Serpe R., Massa E., Mantovani G. (2007). Early epirubicin-induced myocardial dysfunction revealed by serial tissue doppler echocardiography: Correlation with inflammatory and oxidative stress markers. Oncologist.

[5] Deidda M., Madonna R., Mango R., Pagliaro P., Bassareo P.P., Cugusi L., Romano S., Penco M., Romeo F., Mercuro G. (2016). Novel insights in pathophysiology of antiblastic drugs-induced cardiotoxicity and cardioprotection. J. Cardiovasc. Med. (Hagerstown) Spec. Issue Cardiotoxic. Antiblast. Drugs Cardioprot..

[6] Deidda M., Piras C., Bassareo P.P., Cadeddu Dessalvi C., Mercuro G. (2015). Metabolomics, a promising approach to translational research in cardiology. IJC Metab. Endocr..

[7] Deidda M., Piras C., Dessalvi C.C., Locci E., Barberini L., Torri F., Ascedu F., Atzori L., Mercuro G. (2015). Metabolomic approach to profile functional and metabolic changes in heart failure. J. Transl. Med..

[8] Neubauer S. (2017). The failing heart—An engine out of fuel. N. Engl. J. Med..

[9] Osorio J.C., Stanley W.C., Linke A., Castellari M., Diep Q.N., Panchal A.R., Hintze T.H., Lopaschuk G.D., Recchia F.A. (2002). Impaired myocardial fatty acid oxidation and reduced protein expression of retinoid X receptor-alpha in pacing-induced heart failure. Circulation.

[10] Chandler M.P., Kerner J., Huang H., Vazquez E., Reszko A., Martini W.Z., Hoppel C.L., Imai M., Rastogi S., Sabbah H.N. (2004). Moderate severity heart failure does not involve a downregulation of myocardial fatty acid oxidation. Am. J. Physiol Heart Circ. Physiol..

[11] Nascimben L., Ingwall J.S., Lorell B.H., Pinz I., Schultz V., Tornheim K., Tian R. (2004). Mechanisms for increased glycolysis in the hypertrophied rat heart. Hypertension.

[12] Taylor M., Wallhaus T.R., Degrado T.R., Russell D.C., Stanko P., Nickles R.J., Stone C.K. (2001). An evaluation of myocardial fatty acid and glucose uptake using PET with [18F]fluoro-6-thia-heptadecanoic acid and [18F]FDG in patients with congestive heart failure. J. Nucl. Med..

[13] Fillmore N., Lopaschuk G.D. (2013). Targeting mitochondrial oxidative metabolism as an approach to treat heart failure. Biochim. Biophys. Acta.

[14] Chong C.R., Sallustio B., Horowitz J.D. (2016). Drugs that affect cardiac metabolism: Focus on perhexiline. Cardiovasc. Drugs Ther..

[15] Revenco D., Morgan J.P. (2009). Metabolic modulation and cellular therapy of cardiac dysfunction and failure. J. Cell. Mol. Med..

[16] Noordali H., Loudon B.L., Frenneaux M.P., Madhani M. (2018). Cardiac metabolism—A promising therapeutic target for heart failure. Pharmacol. Ther..

[17] Kantor P.F., Lucien A., Kozak R., Lopaschuk G.D. (2000). The antianginal drug trimetazidine shifts cardiac energy metabolism from fatty acid oxidation to glucose oxidation by inhibiting mitochondrial long-chain 3-ketoacyl coenzyme A thiolase. Circ. Res..

[18] Lee L., Horowitz J., Frenneaux M. (2004). Metabolic manipulation in ischaemic heart disease, a novel approach to treatment. Eur. Heart J..

[19] Li Y.J., Wang P.H., Chen C., Zou M.H., Wang D.W. (2010). Improvement of mechanical heart function by trimetazidine in db/db mice. Acta Pharmacol. Sin..

[20] Cheng J.F., Huang Y., Penuliar R., Nishimoto M., Liu L., Arrhenius T., Yang G., O’leary E., Barbosa M., Barr R. (2006). Discovery of potent and orally available malonyl-coa decarboxylase inhibitors as cardioprotective agents. J. Med. Chem..

[21] Wu H., Zhu Q., Cai M., Tong X., Liu D., Huang J., Yang G., Jiang Y. (2014). Effect of inhibiting malonyl-coa decarboxylase on cardiac remodelling after myocardial infarction in rats. Cardiology.

[22] Quigley A.F., Kapsa R.M., Esmore D., Hale G., Byrne E. (2000). Mitochondrial respiratory chain activity in idiopathic dilated cardiomyopathy. J. Card. Fail..

[23] Vega R.B., Huss J.M., Kelly D.P. (2000). The coactivator PGC-1 cooperates with peroxisome proliferator-activated receptor alpha in transcriptional control of nuclear genes encoding mitochondrial fatty acid oxidation enzymes. Mol. Cell. Biol..

[24] Marin-Garcia J., Goldenthal M.J., Moe G.W. (2001). Mitochondrial pathology in cardiac failure. Cardiovasc. Res..

[25] Lehman J.J., Kelly D.P. (2002). Transcriptional activation of energy metabolic switches in the developing and hypertrophied heart. Clin. Exp. Pharmacol. Physiol..

[26] Ventura-Clapier R., Garnier A., Veksler V. (2004). Energy metabolism in heart failure. J. Physiol..

[27] Arany Z., Novikov M., Chin S., Ma Y., Rosenzweig A., Spiegelman B.M. (2006). Transverse aortic constriction leads to accelerated heart failure in mice lacking PPAR-gamma coactivator 1alpha. Proc. Natl. Acad. Sci. USA.

[28] Li P., Luo S., Pan C., Cheng X. (2015). Modulation of fatty acid metabolism is involved in the alleviation of isoproterenol-induced rat heart failure by fenofibrate. Mol. Med. Rep..

[29] Labinskyy V., Bellomo M., Chandler M.P., Young M.E., Lionetti V., Qanud K., Bigazzi F., Sampietro T., Stanley W.C., Recchia F.A. (2007). Chronic activation of peroxisome proliferator-activated receptor-alpha with fenofibrate prevents alterations in cardiac metabolic phenotype without changing the onset of decompensation in pacing-induced heart failure. J. Pharmacol. Exp. Ther..

[30] Akhmedov A.T., Rybin V., Marín-García J. (2015). Mitochondrial oxidative metabolism and uncoupling proteins in the failing heart. Heart Fail. Rev..

[31] Murray A.J., Anderson R.E., Watson G.C., Radda G.K., Clarke K. (2004). Uncoupling proteins in human heart. Lancet.

[32] Wyss M., Kaddurah-Daouk R. (2000). Creatine and creatinine metabolisms. Physiol. Rev..

[33] Liu J., Wang C., Murakami Y., Gong G., Ishibashi Y., Prody C., Ochiai K., Bache R.J., Godinot C., Zhang J. (2001). Mitochondrial ATPase and high-energy phosphates in failing hearts. Am. J. Physiol. Heart Circ. Physiol..

[34] Conway M.A., Allis J., Ouwerkerk R., Niioka T., Rajagopalan B., Radda G.K. (1991). Detection of low phosphocreatine to ATP ratio in failing hypertrophied human myocardium by 31P magnetic resonance spectroscopy. Lancet.

[35] Shen W., Asai K., Uechi M., Mathier M.A., Shannon R.P., Vatner S.F., Ingwall J.S. (1999). Progressive loss of myocardial ATP due to a loss of total purines during the development of heart failure in dogs: A compensatory role for the parallel loss of creatine. Circulation.

[36] Starling R.C., Hammer D.F., Altschuld R.A. (1998). Human myocardial ATP content and in vivo contractile function. Mol. Cell. Biochem..

[37] Beer M., Seyfarth T., Sandstede J., Landschütz W., Lipke C., Köstler H., von Kienlin M., Harre K., Hahn D., Neubauer S. (2002). Absolute concentrations of high-energy phosphate metabolites in normal, hypertrophied, and failing human myocardium measured noninvasively with (31)P-SLOOP magnetic resonance spectroscopy. J. Am. Coll. Cardiol..

[38] Nascimben L., Ingwall J.S., Pauletto P., Friedrich J., Gwathmey J.K., Saks V., Pessina A.C., Allen P.D. (1996). Creatine kinase system in failing and nonfailing human myocardium. Circulation.

[39] Kim T.T., Dyck J.R. (2015). Is AMPK the savior of the failing heart?. Trends Endocrinol. Metab..

[40] Zaha V.G., Young L.H. (2012). AMP-activated protein kinase regulation and biological actions in the heart. Circ. Res..

[41] Sasaki H., Asanuma H., Fujita M., Takahama H., Wakeno M., Ito S., Ogai A., Asakura M., Kim J., Minamino T. (2009). Metformin prevents progression of heart failure in dogs: Role of amp-activated protein kinase. Circulation.

[42] Gundewar S., Calvert J.W., Jha S., Toedt-Pingel I., Ji S.Y., Nunez D., Ramachandran A., Anaya-Cisneros M., Tian R., Lefer D.J. (2009). Activation of amp-activated protein kinase by metformin improves left ventricular function and survival in heart failure. Circ. Res..

[43] Coen M., Holmes E., Lindon J.C., Nicholson J.K. (2008). NMR-based metabolic profiling and metabonomic approaches to problems in molecular toxicology. Chem. Res. Toxicol..

[44] Wishart D.S., Jewison T., Guo A.C., Wilson M., Knox C., Liu Y., Djoumbou Y., Mandal R., Aziat F., Dong E. (2013). HMDB 3.0—The human metabolome database in 2013. Nucl. Acids Res..

[45] Wishart D.S., Knox C., Guo A.C., Eisner R., Young N., Gautam B., Hau D.D., Psychogios N., Dong E., Bouatra S. (2009). HMDB: A knowledgebase for the human metabolome. Nucl. Acids Res..

[46] Gika H.G., Theodoridis G.A., Wilson I.D. (2008). Liquid chromatography and ultra-performance liquid chromatography-mass spectrometry fingerprinting of human urine: Sample stability under different handling and storage conditions for metabonomics studies. J. Chromatogr. A.

[47] Griffin J.L., Atherton H., Shockcor J., Atzori L. (2011). Metabolomics as a tool for cardiac research. Nat. Rev. Cardiol..

[48] Van den Berg R.A., Hoefsloot H.C., Westerhuis J.A., Smilde A.K., van der Werf M.J. (2006). Centering, scaling, and transformations: Improving the biological information content of metabolomics data. BMC Genom..

[49] Andreadou I., Sigala F., Iliodromitis E.K., Papaefthimiou M., Sigalas C., Aligiannis N., Savvari P., Gorgoulis V., Papalabros E., Kremastinos D.T. (2007). Acute doxorubicin cardiotoxicity is successfully treated with the phytochemical oleuropein through suppression of oxidative and nitrosative stress. J. Mol. Cell. Cardiol..

[50] Andreadou I., Papaefthimiou M., Zira A., Constantinou M., Sigala F., Skaltsounis A.L., Tsantili-Kakoulidou A., Iliodromitis E.K., Kremastinos D.T., Mikros E. (2009). Metabonomic identification of novel biomarkers in doxorubicin cardiotoxicity and protective effect of the natural antioxidant oleuropein. NMR Biomed..

[51] Andreadou I., Mikros E., Ioannidis K., Sigala F., Naka K., Kostidis S., Farmakis D., Tenta R., Kavantzas N., Bibli S.I. (2014). Oleuropein prevents doxorubicin-induced cardiomyopathy interfering with signaling molecules and cardiomyocyte metabolism. J. Mol. Cell. Cardiol..

[52] Tan G., Lou Z., Liao W., Zhu Z., Dong X., Zhang W., Li W., Chai Y. (2011). Potential biomarkers in mouse myocardium of doxorubicin-induced cardiomyopathy: A metabonomic method and its application. PLoS ONE.

[53] Cong W., Liang Q., Li L., Shi J., Liu Q., Feng Y., Wang Y., Luo G. (2012). Metabonomic study on the cumulative cardiotoxicity of a pirarubicin liposome powder. Talanta.

[54] Li Y., Ju L., Hou Z., Deng H., Zhang Z., Wang L., Yang Z., Yin J., Zhang Y. (2015). Screening, verification, and optimization of biomarkers for early prediction of cardiotoxicity based on metabolomics. J. Proteome Res..

[55] Schnackenberg L.K., Pence L., Vijay V., Moland C.L., George N., Cao Z., Yu L.R., Fuscoe J.C., Beger R.D., Desai V.G. (2016). Early metabolomics changes in heart and plasma during chronic doxorubicin treatment in B6C3F1 mice. J. Appl. Toxicol..

[56] Yin J., Xie J., Guo X., Ju L., Li Y., Zhang Y. (2016). Plasma metabolic profiling analysis of cyclophosphamide-induced cardiotoxicity using metabolomics coupled with UPLC/Q-TOF-MS and ROC curve. J. Chromatogr. B Anal. Technol. Biomed. Life Sci..

[57] Chaudhari U., Ellis J.K., Wagh V., Nemade H., Hescheler J., Keun H.C., Sachinidis A. (2017). Metabolite signatures of doxorubicin induced toxicity in human induced pluripotent stem cell-derived cardiomyocytes. Amino Acids.

[58] QuanJun Y., GenJin Y., LiLi W., YongLong H., Yan H., Jie L., JinLu H., Jin L., Run G., Cheng G. (2017). Protective effects of dexrazoxane against doxorubicin-induced cardiotoxicity: A metabolomic study. PLoS ONE.

[59] Jensen B.C., Parry T.L., Huang W., Beak J.Y., Ilaiwy A., Bain J.R., Newgard C.B., Muehlbauer M.J., Patterson C., Johnson G.L. (2017). Effects of the kinase inhibitor sorafenib on heart, muscle, liver and plasma metabolism in vivo using non-targeted metabolomics analysis. Br. J. Pharmacol..

[60] Jensen B.C., Parry T.L., Huang W., Ilaiwy A., Bain J.R., Muehlbauer M.J., O’Neal S.K., Patterson C., Johnson G.L., Willis M.S. (2017). Non-Targeted metabolomics analysis of the effects of tyrosine kinase inhibitors sunitinib and erlotinib on heart, muscle, liver and serum metabolism in vivo. Metabolites.

[61] Yoon C.S., Kim H.K., Mishchenko N.P., Vasileva E.A., Fedoreyev S.A., Stonik V.A., Han J. (2018). Spinochrome D attenuates doxorubicin-induced cardiomyocyte death via improving glutathione metabolism and attenuating oxidative stress. Mar. Drugs.

[62] Gramatyka M., Skorupa A., Sokół M. (2018). Nuclear magnetic resonance spectroscopy reveals metabolic changes in living cardiomyocytes after low doses of ionizing radiation. Acta Biochim. Pol..

[63] Madonna R., Cadeddu C., Deidda M., Giricz Z., Madeddu C., Mele D., Monte I., Novo G., Pagliaro P., Pepe A. (2015). Cardioprotection by gene therapy: A review paper on behalf of the working group on drug cardiotoxicity and cardioprotection of the Italian Society of Cardiology. Int. J. Cardiol..

[64] Tocchetti C.G., Cadeddu C., Di Lisi D., Femminò S., Madonna R., Mele D., Monte I., Novo G., Penna C., Pepe A. (2019). From Molecular Mechanisms to Clinical Management of Antineoplastic Drug-Induced Cardiovascular Toxicity: A Translational Overview. Antioxid. Redox Signal..

[65] Cadeddu C., Mercurio V., Spallarossa P., Nodari S., Triggiani M., Monte I., Piras R., Madonna R., Pagliaro P., Tocchetti C.G. (2016). Preventing antiblastic drug-related cardiomyopathy: Old and new therapeutic strategies. J. Cardiovasc. Med. (Hagerstown).

